# Recurrent patellofemoral instability rates after MPFL reconstruction techniques are in the range of instability rates after other soft tissue realignment techniques

**DOI:** 10.1007/s00167-019-05656-3

**Published:** 2019-08-07

**Authors:** Olivier E. Wilkens, Gerjon Hannink, Sebastiaan A. W. van de Groes

**Affiliations:** 1 Department of Orthopedics, Radboud University Medical Center, Nijmegen, The Netherlands; 2 Department of Operating Rooms, Radboud University Medical Center, PO Box 9101, 6500 HB Nijmegen, The Netherlands

**Keywords:** Patellar instability, Recurrent patellar dislocation, MPFL, Medial patellofemoral ligament reconstruction, Skeletally immature, Open physes

## Abstract

**Purpose:**

Recurrent patellofemoral instability is a common knee injury in skeletally immature patients. Many surgical techniques have been described in the literature, all with different success rates. Purpose of this study was to perform a systematic review and meta-analysis of the available literature to assess recurrent patellofemoral instability rates after surgical treatment using MPFL reconstruction techniques and other soft tissue realignment techniques in skeletally immature patients.

**Methods:**

PubMed, Embase, Web of Science, and The Cochrane Library were searched to identify all original articles concerning the surgical treatment for patellofemoral instability in skeletally immature patients and that reported post-operative recurrent patellofemoral instability rates. Subsequently a risk of bias assessment was conducted and a meta-analysis was performed on reported post-operative recurrent patellofemoral instability rates after MPFL reconstruction techniques and other soft tissue realignment techniques.

**Results:**

Of the 21 eligible studies (448 knees in 389 patients), 10 studies reported on MPFL reconstruction techniques using different grafts and fixation techniques and 11 reported on other soft tissue realignment procedures. In total, 62 of the 448 (13.8%) treated knees showed recurrent patellofemoral instability during follow-up. The overall pooled recurrent patellofemoral instability rate was estimated to be 0.08 (95% CI 0.02–0.16). For MPFL reconstruction techniques, the pooled recurrent patellofemoral instability rate was estimated to be 0.02 (95% CI 0.00–0.09). For the other soft tissue realignment techniques, the pooled rate was estimated to be 0.15 (95% CI 0.04–0.31).

No statistically significant difference in recurrent patellofemoral instability rates between MPFL reconstruction techniques and other soft tissue realignment techniques were found (n.s.). There was a large variation in treatment effects over different settings, including what effect is to be expected in future patients.

**Conclusion:**

This systematic review and meta-analysis found that recurrent patellofemoral instability rates after MPFL reconstruction techniques are in the range of instability rates after other soft tissue realignment techniques. The clinical relevance of this study is that it provides clinicians with the best currently available evidence on recurrent patellofemoral instability rates after surgical treatment for patellofemoral instability in skeletally immature patients.

**Level of evidence:**

IV.

## Introduction

Patellofemoral dislocation or subluxation is a common knee injury in children and young adolescents. The overall annual incidence of patellar dislocation has been estimated to be 23.2 per 100,000, with a highest annual incidence among adolescents aged 14 to 18 years of 147.7 per 100,000 [33]. However, as most epidemiological studies focus on the adult population, the exact numbers for skeletally immature patients with patellofemoral instability are still unknown [36]**.** The risk of recurrent instability reported in literature varies widely, ranging from 11 to 60% after primary dislocation [17, 21, 26, 32].

The pathomechanism of patellofemoral instability is complex and often multifactorial. The osseous anatomy of the entire femur, in both torsion and trochlea shape, is often abnormal and the rotation of the tibia and the ligamentous stability (i.e. laxity) of the knee have been reported to be important predisposing factors to develop patellofemoral instability or pain [26, 31, 37, 39].

After first time dislocation, conservative treatment is indicated, whereas surgery is the treatment of first choice in case of recurrence [29].

In adults, the main surgical goal is to restore the bony mismatch in the knee, for instance, by performing a tibial tubercle transfer or trochleoplasty. There is still much controversy in the current literature as to what extend and degree of bone pathology requires correction in addition to a MPFL reconstruction [29].

However, these bony surgical procedures are generally not indicated in skeletally immature patients due to the risk to damage an open growth plate, and the subsequent development of bony deformities. Nelitz et al. [23] recently showed that for selected adolescent patients with high-grade trochlear dysplasia, trochleoplasty can be safely performed up to 2 years before the projected end of growth. However, so far, soft tissue (balancing) or realignment techniques are the generally preferred operative options for skeletally immature patients [5, 34].

In the past century many realignment techniques, such as the (Roux-) Goldthwait, the Galeazzi semitendinosus tenodesis, the lateral retinaculum release, the medial retinaculum reefing/imbrication or any combined procedures have been described [2-4, 7, 11, 13, 16, 27, 31, 36]. The success rates of these techniques vary widely, and none of these techniques has been shown to be superior to the other**.** In the last decade, several studies have shown the importance of the medial patellofemoral ligament (MPFL) as a medial restraint against lateral patellar displacement in early knee flexion, and several promising MPFL reconstruction techniques, with different grafts and/or fixation points, have been described in skeletally immature patients [1, 8, 15, 17, 21, 22, 27, 42].

The purpose of this systematic review and meta-analysis was to identify all available evidence on recurrent patellofemoral instability rates after MPFL reconstruction techniques and other soft tissue realignment techniques in skeletally immature patients. The results of this study will provide clinicians with the best currently available evidence on recurrent patellofemoral instability rates after surgical treatment for patellofemoral instability in skeletally immature patients, can be helpful in the process of deciding whether or not to perform such a procedure, and can be used to better inform patients about the advantages and disadvantages of different procedures.

## Materials and methods

This systematic review investigates recurrent patellofemoral instability rates after MPFL reconstruction techniques and other soft tissue realignment techniques in skeletally immature patients. The inclusion criteria and method of analysis were specified in advance and documented in a protocol (PROSPERO CRD42017069706) and the study is reported according to PRISMA guidelines [20].

### Search strategy and selection

Pubmed, Embase, Web of Science, and The Cochrane Library were searched (last search performed May 8, 2019) for articles concerning randomized controlled trials (RCTs), quasi-randomized trials and all observational studies. The search strategy, composed of three elements (patella, instability, and skeletally immature), was developed in collaboration with information specialists from the medical library of the Radboud university medical center Nijmegen, the Netherlands. The detailed search strategy is provided in Appendix 1.

Reference lists of the selected relevant (review) papers were screened for potentially missed papers, and no restrictions in publication date were imposed. Only articles in English, German, French and Dutch were selected. Search results were imported in EROS (Early Review Organizing Software, developed by Institute of Clinical Effectiveness and Health Policy, Buenos Aires, Argentina) to remove duplicates, and randomly allocate references to two independent reviewers responsible for screening, selection and data extraction (OW, SvdG). Discrepancies were resolved by discussion and if necessary a third reviewer was consulted (GH).

Initially, during the screening phase, primary studies evaluating any treatment for recurrent patellofemoral instability in skeletally immature patients were selected based on their title and abstract only. Review articles, letters, conference abstracts were excluded. In addition, articles with congenital (syndromic) or primary/acute patellar instability were also excluded. In the event that there was insufficient information to make a valid judgment, the whole publication was evaluated. Full-text copies of all publications eligible for inclusion were subsequently assessed and included when they met our prespecified inclusion criteria: (1) randomized controlled trials (RCTs), quasi-randomized trial, or other observational study design; (2) skeletally immature patients [defined as human individuals with open physes (radiological) or age ≤ 12 years (girls) or ≤ 14 years (boys)]; (3) description of (semi-)quantitative outcome measures related to recurrent patellofemoral instability (defined as repeated dislocation or subluxation of the patella).

### Data extraction

Next to bibliographic details, data on study design, number of patients, number of knees, type of intervention, and outcome measures were extracted. Attempts were made to obtain original data by contacting authors if results were presented incomplete or graphically only. If not otherwise possible, graphically presented data were converted to numerical data using digital ruler software (Plot Digitizer, University of South Alabama, USA).

### Risk of bias

The quality of the included studies was assessed using the risk of bias in non-randomized studies of interventions (ROBINS-I) assessment tool by two reviewers (OW, SvdG) independently. The ROBINS-I tool uses the Cochrane-approved risk of bias approach and focuses on risk of bias due to the counterfactual and consequently articulates limitations in the assessed studies [38].

### Statistical analysis

Statistical analyses were performed using R version 3.6.0 (R Foundation for Statistical Computing, Vienna, Austria) with package 'meta'. Whenever three or more studies per surgical technique (MPFL reconstruction or other soft tissue realignment techniques) reported on recurrent patellar instability, we included these studies in our meta-analysis. Studies with ≤ 3 patients were considered case reports and not included in the meta-analysis. Despite anticipated heterogeneity, the individual study proportions were pooled. Pooled estimates of proportions with their corresponding 95% confidence intervals (CIs) were calculated using Freeman-Tukey double arcsine transformation within a random effects model framework. Heterogeneity of combined study results was assessed by *I*^2^, and its connected chi-square test for heterogeneity were calculated. Restricted maximum likelihood was used to estimate the variance in heterogeneity. 95% prediction intervals (PIs) were calculated to present the expected range of true effects in similar studies [12]. Publication bias was addressed by means of a funnel plot, if at least 15 studies could be included [35].

## Results

**Fig. 1:**
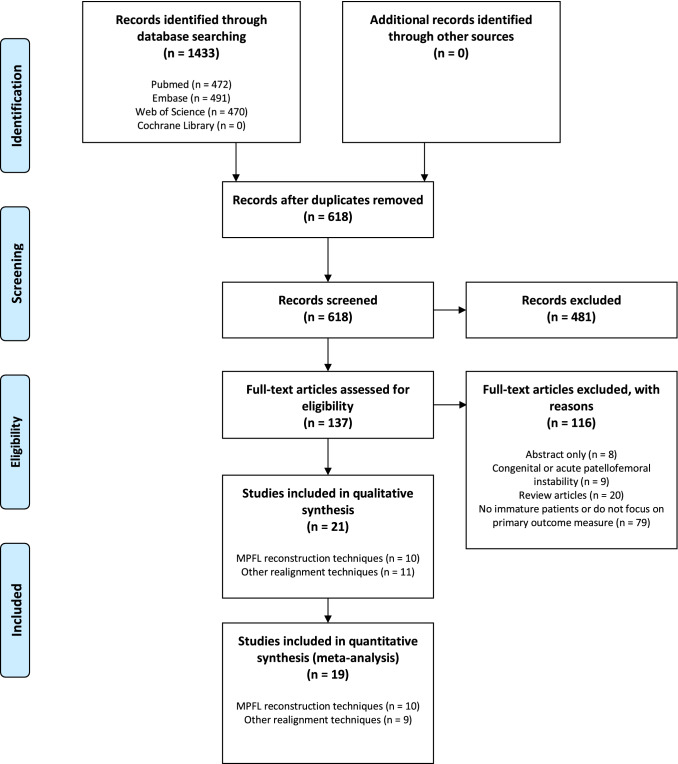
PRISMA flowchart of search results

The search strategy retrieved 1433 records. The subsequent selection procedure resulted in 21 eligible articles. A flow chart of the study selection process is presented in Fig. 1.

Of the 21 eligible articles (448 knees in 389 patients), 10 studies reported on MPFL reconstruction techniques using different grafts and fixation techniques and 11 reported on other soft tissue realignment procedures. In total, 62 of the 448 (13.8%) treated knees showed recurrent patellofemoral instability during follow-up. All 21 studies reported on different surgical techniques or combinations of surgical techniques. There was a large variation in reported recurrent instability rates, varying between 0 and 38% for MPFL reconstruction techniques and between 0 and 82% for other soft tissue realignment techniques. The characteristics of all included studies are summarized in Table 1. Mean follow-up ranged between 17.7 months and 7.4 years and between 12 months and 13.5 years for MPFL reconstruction techniques and other soft tissue realignment techniques, respectively.

**Table 1:** Characteristics of included studies

Author	Study design	Type of treatment	Patients (knees)	Sex (female: male)	Age (years)	Follow-up	Recurrent patellofemoral instability rate	Kujala	Lysholm	Tegner	Insall-salavti ratio	Sulcus angle	Trochlear dysplasia classification
MPFL reconstruction techniques													
Abouelsoud [1]	Prospective case series	Anatomic MPFL reconstruction with no hardware fixation	16 (16)	11:5	11.5 (8–15)	29.25 (24–34) months	0/16 (0%)	Pre: 56 (49–61) Post: 94 (90–99)		Pre: 4.5 (4–7) Post: 5.25 (4–7)	Pre: Post: 1 (1–1.19)		11× mild dysplasia 5× moderate dysplasia
Deie [8]	Retrospective cohort	MPFL reconstruction with the semitendinosus tendon	4 (6)	2:2	8.5 (6–10)	7.4 (4.8–10) years	0/6 (0%)	Pre: Post: 96.3 (89–100)			Pre: Post: 1.4 ± 0.1	Pre: Post: 153.2 ± 2.7	
Kumahashi [15]	Retrospective cohort	MPFL reconstruction: A “sandwich” method: double–stranded semitendinosus autograft and titanium interference anchor system	5 (5)	3:2	13.6 (11–15)	27.8 (24–36) months	0/5 (0%)	Pre: 67.4 ± 12.6 Post: 95.4 ± 3.2	Pre: 64.4 ± 14.1 Post: 96.0 ± 2.2		Pre: 1.2 ± 0.2 Post: 1.2 ± 0.2	Pre: 151.3 ± 15.1 Post: 150.1 ± 16.4	
Lind [17]	Case–control study	MPFL reconstruction with gracilis tendon autograft	20 (24)	11:9	12.5 (8–16)	39 (17–72) months	9/24 (38%)	Pre: 61 ± 13 Post: 1 year: 81 ± 16 Final: 71 ± 15					4× Dejour type A 10× Dejour type B 10× Dejour type C/D
Matuszewski [19]	Randomized controlled trial	MPFL recontruction with cadaver fascia lata allograft (a) MPFL reconstruction with gracilis tendon autograft (b)	22 (22) 22 (22)	12:10 15:7	15.00 (13–17) 14.95 (13–16)	24 (18–30) months 24 (18–30) months	1/22 (4.5%) 0/22 (0%)	Pre: 73.91 (55–86) Post: 94.50 (88–100) Pre: 70.77 (48–90) Post: 94.32 (87–100)					
Nelitz [21]	Case series	Anatomic reconstruction of the MPFL, with gracilis tendon	21 (21)	6:15	12.2 (10.3–13.9)	2.8 (2.0 –3.6) years	0/21 (0%)	Pre: 72.9 (37–87) Post: 92.8 (74–100)		Pre: 6.0 (3–9) Post: 5.8 (3–9)	Pre: 1.2 (1.0–1.3) Post:		1× Dejour type A 10× Dejour type B 4× Dejour type C 6× Dejour type D
Nelitz [22]	Prospective cohort	Anatomic reconstruction of the MPFL, with a superficial quadriceps tendon graft	25 (25)	16:9	12.8 (9.5–14.7)	> 2 years	0/25 (0%)	Pre: 63 (44–81) Post: 89 (77–100)		Pre: 4 (3–8) Post: 5 (3–8)	Pre: 1.2 (1.0–1.3) Post:		15× Dejour type A 10× Dejour A/B
Pesenti [28]	Retrospective cohort	MPFL with hamstring graft	25 (27)	19:6	13.8 ± 2.5	41.1 ± 13.5 months	1/27 (3.7%)	Pre: Post: 95.3					11× trochlear dysplasia
Uppstrom [40]	Retrospective cohort	MPFL reconstruction with hamstring graft and fixation with screws	49 (54)	30:19	13.3 ± 1.6	2.4 (0.5–8.0) years	5/54 (9.3%)						
Yercan [42]	Retrospective cohort	MPFL reconstruction using a free semitendinosus autograft + tenodesis to the adductor magnus tendon	3 (4)	3:0	8.7 (5–13)	17.7 (15 – 20) months	0/4 (0%)	Pre: 36 (35–38) Post: 89.5 (87–92)					
Other realignment techniques													
Benoit [2]	Retrospective cohort	Distal advancement of the patella, lateral release and advancement of VMO	8 (12)	4:4	10.3 (7–14)	13.5 (11–16) years	1/12 (8.3%)	Pre: Post:	Pre: Post: 98 (95–100)	Pre: Post:	Pre: Post:	Pre: < 10 years 164.6 (158–169) > 10 years 156.9 (153 –159) Post: < 10 years 141 (137– 142) > 10 years 150.4 (149–143)	Patellofemoral dysplasia was present in each affected knee
Biglieni [3]	Case series	Goldthwait procedure + lateral release	19 (20)	11:8	13.4 (11.2–15.1)	6.8 (3–10) years	2/20 (10%)						
Bonnard [4]*	Case series	Goldthwait procedure	24 (40)	20:4	7–15 (13)	36 (14 –78) months	3/40 (7.5%)					Pre: 12× < 135 7× 135–140 21× > 140 Post: 142.5 ± 13	
Cootjans [7] †	Retrospective cohort	Medial imbrication (a)	12 (17)	9:3	12 ± 4	3.2 years	5/17 (29%)		Pre: Post: 92.7 ± 6	Pre: Post: 5.25 ± 2.0			
		Medial imbrications + Roux procedure (b)	12 (14)	9:3	13 ± 3	5 years	1/14 (7%)		Pre: Post: 70.1 ± 17	Pre: Post: 3.5 ± 1.8			
Grannatt [11]	Retrospective case series	Galeazzi Semitendinosus tenodesis + lateral release ( + 21/34 medial reefing)	28 (34)	19:9	11.1 (4.5–15.8)	70 (27–217) months	28/34 (82%)	Pre: Post: 79 (no range)					
Joo [13]#	Case report	Four–in–one: lateral release, proximal ‘tube’ realignment of the patella, semitendinosus tenodesis and patellar tendon transfer	2 (3)	2:0	6.2 (5.4–6.8)	54.5 (21–66) months	0/3 (0%)	Pre: Post: 97.3 (96–98)				Pre: Post: 147.5 (142.2–156.1)	Pre–operative: all Dejour type C Final follow–up: all Dejour type A
Letts [16]	Retrospective cohort	Semitendinosus transfer to the patella + lateral retinaculum release + capsular tightening	22 (26)	19:3	14.3 (8.9–17.8)	3.2 (2–7.3) years	2/26 (8%)		Pre: Post: 68 (35–93)				
Malagelada [18]	Retrospective cohort	4–in–1 procedure (lateral release, medial reefing, Insall tube realignment and Roux Goldtwait patella ligament transfer)	12 (16)	8:4	12.6 (9–16)	36 (36–98) months	3/16 (19%)	Pre: Post: 83.4 ± 11.47			Pre: 1.2		Dysplastic throchlea present in 81%
Pesenti [27]$	Retrospective cohort	Medial transposition of the extensor apparatus and MPFL tensioning (7 × )	11 (13)	3:8	11.7 (8–14)	6.1 (5–8) years	0/13 (0%)					Pre: 161 (140–175) Post: 135 (121–150)	All Dejour type B
Ronga [31]	Prospective cohort	Lateral release, vastus medialis muscle advancement and transfer of the medial third of the patella tendon to the medial collateral ligament	25 (25)	7:18	13.5 ± 3.8	3.8 (2.5–6) years	1/25 (4%)	Pre: 52.4 ± 12.7 Post: 93.8 ± 14.2			Pre: 1.04 ± 0.2 Post: 1.02 ± 0.3		
Sugimoto [36]	Case report	1 Roux–Goldthwait procedure and 1 lateral release and medial capsular reefing	2 (2)	1:1	11 12	18 months 12 months	0/1 (0%) 0/1 (0%)					Pre: 134 Post: 119 Pre: 147 Post: 138	

^&^ Matuszewski [19]; both RCT arms were included as separate groups in the analysis

*Bonnard et al. 1990; 9 patients with traumatic patellar instability included

^†^ Cootjans et al. [7]; both types of treatment were included as separate groups in the analysis

^#^ Joo et al. [13]; reported 6 knees in 5 patients, manually deleted 2 patients with Down syndrome and 1 patient with William’s syndrome

^$^ Pesenti et al. [28]; reported 27 knees in 23 patients, manually deleted all skeletally mature patients and patients with Down or Kabuki syndrome

### Risk of bias and quality of reporting

The results of the quality assessment of all included studies are presented in Table 2. There was a considerable risk of bias in most of the included studies and the methodological quality was rated “serious” to “critical”. None of the included articles were randomized nor blinded.

**Table 2:** ROBINS-I Risk of bias assessment

	Domain 1: confounding	Domain 2: selection of participants	Domain 3: classification of intervention	Domain 4: deviation from interventions	Domain 5: missing data	Domain 6: measurement of outcomes	Domain 7: Selection of reported results	ROBINS-I overall
MPFL reconstruction techniques								
Abouelsoud [1]	^Ŧ^0	3	2–3	^Ŧ^0	3	3	^Ŧ^0	Serious
Deie [8]	^Ŧ^0	3	3	^Ŧ^0	2	3	^Ŧ^0	Serious
Kumahashi [15]	^Ŧ^0	3	2	2–3	2	3	^Ŧ^0	Moderate–serious
Lind [17]	4	3	3	^Ŧ^0	3	3	^Ŧ^0	Serious
Matuszewski [19]	1	1	1	1	1	1	1	Low
Nelitz [21]	^Ŧ^0	3	2–3	^Ŧ^0	2	3	^Ŧ^0	Serious
Nelitz [22]	^Ŧ^0	2–3	2–3	2–3	2	3	2	Moderate–serious
Pesenti [28]	^Ŧ^0	3	3	3	3	3	^Ŧ^0	Serious
Uppstrom [40]	Ŧ0	3	2	2	2	2	2	Moderate–serious
Yercan [42]	^Ŧ^0	3–4	4	^Ŧ^0	3	^Ŧ^0	^Ŧ^0	Serious–critical
Other realignment techniques								
Benoit [2]	^Ŧ^0	3	2–3	^Ŧ^0	2	3	2–3	Moderate–serious
Biglieni [3]	^Ŧ^0	4	4	^Ŧ^0	3–4	3	^Ŧ^0	Serious–critical
Bonnard [4]	^Ŧ^0	3	3	^Ŧ^0	3	3	^Ŧ^0	Serious
Cootjans [7]	^Ŧ^0	4	4	^Ŧ^0	4	4	^Ŧ^0	Serious–critical
Grannatt [11]	2–3	3	3	3–4	3	3	3	Serious
Joo [13]	^Ŧ^0	^Ŧ^0	4	3	3	^Ŧ^0	^Ŧ^0	Serious
Letts [16]	^Ŧ^0	3	3	2–3	2–3	3	3	Serious
Malagelada [18]	Ŧ0	3	2	2	2	2	2	Moderate–serious
Pesenti [27]	^Ŧ^0	3–4	3–4	2–3	2	3	^Ŧ^0	Serious
Ronga [31]	^Ŧ^0	3	2–3	^Ŧ^0	3	3	^Ŧ^0	Serious
Sugimoto [36]	^Ŧ^0	4	^Ŧ^0	4	^Ŧ^0	4	4	Critical

Risk of bias assessment: 0 No information; 1 low; 2 moderate; 3 serious; 4 critical

Ŧ0 (no information) was assessed as equivalent to “Serious” (3)

### Results of studies included in the meta-analysis

Ten studies reporting on MPFL reconstruction techniques [1, 8, 15, 17, 19, 21, 22, 28, 40, 42], and nine studies reporting on other soft tissue realignment techniques [2-4, 7, 11, 16, 18, 27, 31] were included in the meta-analysis (Fig. 1).

**Fig. 2:**
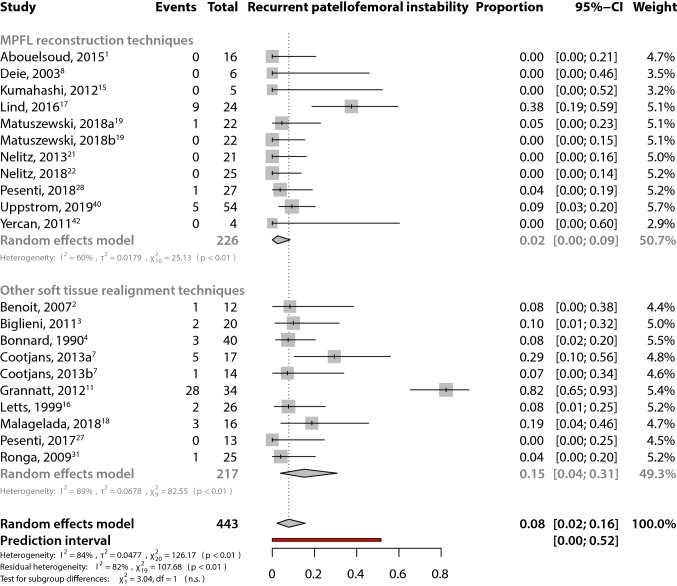
Forest plots of the included studies using the MPFL reconstruction techniques and other soft tissue realignment techniques. Forest plots display the proportion of complications, 95% confidence interval and the relative weight of the individual studies. The diamond indicates the pooled estimate and its 95% confidence interval. The red bar indicates the 95% prediction interval. Prediction intervals illustrate which range of true effects expected to occur in similar studies in future settings. Matuszewski et al. [19] reported a randomized controlled trial (RCT) comparing two different MPFL reconstruction techniques using **a** fascia lata allograft, and **b** gracilis tendon autograft. Both RCT arms were included as separate groups in the analysis. Cootjans et al. [7] reported a retrospective cohort study consisting of two cohorts using **a** medial imbrication alone, and **b** medial imbrication combined with a Roux procedure. Both cohorts were included as separate groups in the analysis

The overall pooled recurrent patellofemoral instability rate was estimated to be 0.08 (95% CI 0.02–0.16) (Fig. 2). For MPFL reconstruction techniques, the pooled recurrent patellofemoral instability rate was estimated to be 0.02 (95% CI 0.00–0.09) (Fig. 2). For the other soft tissue realignment techniques, the pooled rate was estimated to be 0.15 (95% CI 0.04–0.31) (Fig. 2). The 95% PIs reflect the variation in treatment effects over different settings, including what effect is to be expected in future patients, such as the patients that a clinician is interested to treat. The PIs reflect the large heterogeneity in both the MPFL reconstruction techniques [95% PI, 0.00–0.27 (heterogeneity: *I*^2^ = 60%; *p* < 0.01)] and the other soft tissue realignment techniques [95% PI, 0.00–0.77 (heterogeneity: *I*^2^ = 89%; *p* ≤ 0.01)]. No statistically significant difference in recurrent patellofemoral instability rates between MPFL reconstruction techniques and other soft tissue realignment techniques were found (*χ*^2^ = 3.04; n.s.).

### Results of studies not included in the meta-analysis

All studies on MPFL reconstruction techniques were included in the meta-analysis. Sugimoto et al. [36] and Joo et al. [13] were not included in the meta-analysis for the other soft tissue realignment techniques as these were considered case-reports. Sugimoto et al. [36] performed a Roux-Goldthwait procedure and a lateral release combined with a medial capsular reefing on two patients, and Joo et al. [13] performed a four-in-one procedure: a lateral release, proximal ‘tube’ realignment of the patella, semitendinosus tenodesis and patellar tendon transfer on two patients. Both studies reported 0% recurrent patellofemoral instability rates.

### Publication bias

Due to the low number of studies that were included in the meta-analyses the possible presence of publication bias could not reliably be assessed.

## Discussion

The most important finding of this systematic review and meta-analysis was that recurrent patellofemoral instability rates using MPFL reconstruction techniques were in the range of instability rates after other soft tissue realignment techniques. There was a large variation in both surgical techniques and reported recurrent instability rates, varying between 0 and 38% for MPFL reconstruction techniques and between 0 and 82% for other soft tissue realignment techniques.

In the MPFL reconstruction techniques, 9 out of 10 studies reported low post-operative recurrent patellofemoral instability rates. Only Lind et al. [17] reported a 38% (9/24 knees) recurrent patellofemoral instability rate at final follow-up. They reported on patients with various degrees of patellofemoral dysplasia, which may explain the high recurrent patellofemoral instability rate. However, they could not find an association between the high degree of trochlea dysplasia (grade C and D) and the redislocation rates [17]. Abouelsoud et al. [1] reported no recurrent patellofemoral instability after MPFL reconstruction, but five cases could be described as infrequent subluxation episodes. Patients with severe trochlear dysplasia were excluded in their study.

In the other soft tissue realignment techniques, 8 out of 11 studies reported post-operative recurrent instability rates less than or equal to 10%. Two studies reported rates higher than 20%. Cootjans et al. [7] reported a recurrent instability rate of 29%, while Grannatt et al. [11], reported an 82% rate at final follow-up. An explanation for the poor results in Grannatt et al. [11] could be the long duration of follow-up. Patients had a minimum follow-up of 2-year with a mean follow-up of 5.8 years (range 27–217 months). They concluded that the Galeazzi procedure may be associated with higher rates of recurrent instability and more debilitated knee function than previously appreciated. Cootjans et al. [7] reported a very low response rate on the questionnaire and analyzed and reported the data based on the available questionnaires.

Despite the more anatomical nature of a MPFL reconstruction, in the present study no clear advantage of MPFL reconstruction techniques over other soft tissue realignment techniques was found as the confidence intervals were overlapping. One of the most important reasons for MPFL reconstruction failure in young patients is severe trochlear dysplasia [24]. Generally, this is not addressed until patients have closed physes. Since younger patients have often more severe dysplasia or rotational deformities, this might explain the similar recurrence rates in both groups. Despite severe trochlear dysplasia or increased femoral anteversion, technical errors (e.g. non-anatomic bone tunnels or overtensioning of the graft) are also a common cause for MPFL reconstruction failure [25]. An MPFL reconstruction remains a challenging procedure in young patients, particularly in those with additional bony deformities with attribute to patellar instability, and should, therefore, be performed by experienced surgeons.

Some limitations of this study have to be discussed. First, the definition of recurrent patellofemoral instability, that is redislocation or subluxation, is arguable and might differ between clinicians and/or patients. In addition, relying on patient reported recurrent patellofemoral instability may result in not all occurrences being reported. Second, there is no clear consensus on indication for the use an MPFL reconstruction techniques or other soft tissue realignment techniques, which hampers a comparison between studies and/or techniques. The presented recurrent patellofemoral instability rates for different techniques should be interpreted in the context of the individual studies that have been published, including exact indication for surgery, duration and severity of symptoms, and patient factors. Predisposing factors, such as increased Q-angle and TT-TG in combination with all the limitations of soft tissue procedures could also explain a high recurrent patellofemoral instability rate, but these are unknown for all individual patients included in the studies. Third, almost all studies were retrospective or prospective case series and publication bias may be present since “negative” results of case series of surgical procedures are less likely to be submitted for publication. None of the studies were randomized nor blinded and there was a considerable risk of bias in most of the included studies.

Skeletally immature patients have the unique advantage that their bones are capable of remodeling after injury or a surgical intervention. Sugimoto et al. [36] described a decrease in sulcus angle after surgery, suggesting the femoral trochlea was deepened and remodelled due to a more centralized patella. Joo et al. [13] concluded an improvement in development of the femoral trochlea after surgery. In contrast, Rajdev et al. [30] showed no remodeling of the femoral trochlea after patellar stabilization. However, in that study the mean age was 14.7 years and therefore most of the patients were after their growth spurt. In the same study, the age of 10 years is indicated as an important age after which trochlear remodeling is limited. It can be assumed that with a skeletal age of 12 years for girls and 14 years for boys, the main growth spurt is over and there is only residual growth after that and no clear remodeling is to be expected [23]. This conclusion is also supported by Fu et al. [9], who showed trochlear remodeling in a patient population ranging from 7–11 years of age. Therefore, the relation between soft tissue realignment and remodeling seems to be clearly related to the start of the growth spurt of a patient. Evidence is still very limited due to lack of information in most studies. Nevertheless, trochlear remodeling due to patella realignment surgery is a topic which needs to be addressed more in detail, since it has not been well described in the pediatric orthopedic literature yet.

Many different surgical techniques on skeletally mature patients have been reported. These surgical techniques may be used on skeletally immature patients, however, should be possibly modified and further studied before use in this young patient population [6, 10, 14, 41].

The clinical relevance of this study is that it provides clinicians with the best currently available evidence on recurrent patellofemoral instability rates after surgical treatment for patellofemoral instability in skeletally immature patients. This can be helpful in the process of deciding whether or not to perform such a procedure, and can be used to better inform patients about the advantages and disadvantages of different procedures.

## Conclusion

This systematic review and meta-analysis found that recurrent patellofemoral instability rates after MPFL reconstruction techniques are in the range of instability rates after other soft tissue realignment techniques.
